# Rural–Urban Disparities in Access to Medicaid-Contracted Pharmacies in Washington State, 2017

**DOI:** 10.5888/pcd17.200066

**Published:** 2020-08-20

**Authors:** Janessa M. Graves, Demetrius A. Abshire, Megan Undeberg, Laura Forman, Solmaz Amiri

**Affiliations:** 1 Washington State University, College of Nursing, Spokane, Washington; 2 Washington State University, Honors College, Pullman, Washington; 3 University of South Carolina, College of Nursing, Columbia, South Carolina; 4 Washington State University, College of Pharmacy, Spokane, Washington; 5 Washington State University, Elson S. Floyd College of Medicine, Spokane, Washington

## Abstract

**Introduction:**

Community retail pharmacies offer multiple public health services to meet the health care needs of medically underserved rural communities. Many rural residents are enrolled in Medicaid insurance, and it is important that pharmacies contract with Medicaid to meet the health care needs of these people. The objective of this study was to evaluate disparities in access to Medicaid-contracted pharmacies across the rural–urban continuum in Washington State.

**Methods:**

We linked data on licensed community retail pharmacies in Washington State in 2017 to lists of state Medicaid-contracted pharmacies. We classified pharmacies as being located in small rural, large rural, suburban, and urban areas by using rural–urban commuting area (RUCA) codes. We evaluated the likelihood of zip code–level access to at least 1 pharmacy that was contracted with a Medicaid insurance plan across the rural–urban continuum by using descriptive statistics and modified Poisson regression models, adjusted for zip code–level community characteristics.

**Results:**

Of 1,145 pharmacies in our study sample, 8.4% (n = 96) were not contracted with a Medicaid plan. Compared with urban core zip codes, small rural zip codes (adjusted relative risk [ARR] = 0.64; 95% CI, 0.46–0.91) and large rural zip codes (ARR = 0.68; 95% CI, 0.49–0.95) were significantly less likely to have access to a Medicaid-contracted pharmacy. Suburban zip codes did not differ significantly from urban core areas in their access to Medicaid-contracted pharmacies.

**Conclusion:**

In Washington State, the likelihood of access to a Medicaid-contracted pharmacy decreased significantly as rurality increased. Policy efforts should aim to improve access for Medicaid enrollees, especially those outside urban centers.

**Summary**

**What is already known on this topic?**

Community retail pharmacies provide prescription services, as well as health promotion and disease management services, such as immunizations, rapid influenza screening, cholesterol testing, blood pressure management, blood glucose monitoring, and substance use treatment.

**What is added by this report?**

Areas outside urban core centers in Washington State were significantly less likely to have access to a pharmacy contracted with Medicaid insurance. Disparities in access to pharmacy care exist for Medicaid recipients across the rural–urban continuum.

**What are the implications for public health practice?**

Ensuring that rural residents have access to pharmacies that contract with Medicaid is important for improving access to health care in rural areas. Public health professionals should advocate for policies to ensure such access.

## Introduction

Pharmacists serve an essential role in the provision of community-based services in the United States. Besides traditional services such as dispensing prescription and over-the-counter medications, pharmacists’ roles now include extended services such as immunizations, rapid influenza screening, wellness testing, chronic disease screening and management, health education, medication monitoring and reviews, emergency contraception, smoking cessation, substance use treatment, and prevention of hospital readmission (1–4). As a testament to the importance of their role and the array of services they provide, pharmacists’ contributions have been recognized as critical elements in the prevention and management of chronic diseases in the United States (5)

Pharmacists’ expanded roles have implications for the provision of health care services in rural areas. Rural stakeholders have identified access to quality health care services as the top rural health priority of the decade, along with many other priorities pertaining to chronic conditions and behavioral risk factors (6) that pharmacists are capable of addressing. However, the closure of rural pharmacies is negatively affecting access to health services in rural areas. From 2003 to 2018, the number of independently owned pharmacies in rural areas of the United States decreased by 16% (7). Among 119 community retail pharmacies that closed from 2006 through 2010, thirty-one were in rural communities that had no other health care provider, and 17% of these were in remote rural areas (8). Pharmacy closures are particularly concerning for the nearly 3 million people who live in rural communities that have a single independently owned pharmacy (9). This decreasing number of rural pharmacies creates pharmacy deserts (10), requiring rural residents to travel farther distances for services that were once nearby and mitigating potential gains in access brought about by the expanded role of pharmacies in delivering rural community health services.

Compared with urban residents, rural residents tend to be older, have lower income, and have less education; are less likely to be employed; and are more likely to have a disability (11–13). Rural residents are also less likely to have private health insurance and, therefore, rely more often on public sources of insurance (12,13). The probability of having Medicaid as a result of the Patient Protection and Affordable Care Act’s Medicaid expansion increased more for rural childless adults than for urban childless adults (14), and the effect of expansion on reducing uninsurance was 68% higher in rural areas than in urban areas (15). Medicaid expansion also decreased the percentage of infants born into rural households with no insurance, but this decrease was smaller than the decrease among urban households (16). Pharmacies are not, however, mandated to have a contract with Medicaid prescription insurance. Many pharmacies may not be contracted with Medicaid because of low reimbursement rates, which are contractually established and vary by state (17). Consequently, increasing Medicaid coverage does not unequivocally translate to improved access to pharmacy services.

Because community retail pharmacies may be widely dispersed in areas of low population density, rural residents with Medicaid insurance may have a greater burden than their urban counterparts in accessing timely, affordable medications and other pharmacists’ or pharmacy-related services. Lack of access to pharmacy care can affect a patient’s ability to adhere to prescribed medical regimens (18–20), in addition to restricting access to public health services provided by pharmacies. These services are particularly needed in rural areas, because the prevalence of many chronic conditions and associated risk factors is higher in rural areas than in urban areas (21–24), and many rural areas have seen a loss of general health services.

Washington State is 1 of 5 states in the Pacific Census division, the census division with the highest percentage (~92%) of the population living in urban areas (25). Given the relatively small proportion of rural-dwelling residents in the Pacific Census division, communities of rural-dwelling residents often garner less attention from policy makers and receive fewer state and federal resources to address the health needs of their populations across wide geographical areas. Community pharmacies that contract with Medicaid are an important health care resource for residents of rural communities in Washington State. The objective of this study was to evaluate disparities in access to Medicaid-contracted pharmacies across the rural–urban continuum in Washington State.

## Methods

This cross-sectional study involved merging of pharmacy licensing data and contracted pharmacy lists from Medicaid insurance plans in Washington State. The study was conducted from August 2017 through October 2018 and did not involve human subjects or records; institutional review board approval was not required. 

### Data sources

**Figure 1:**
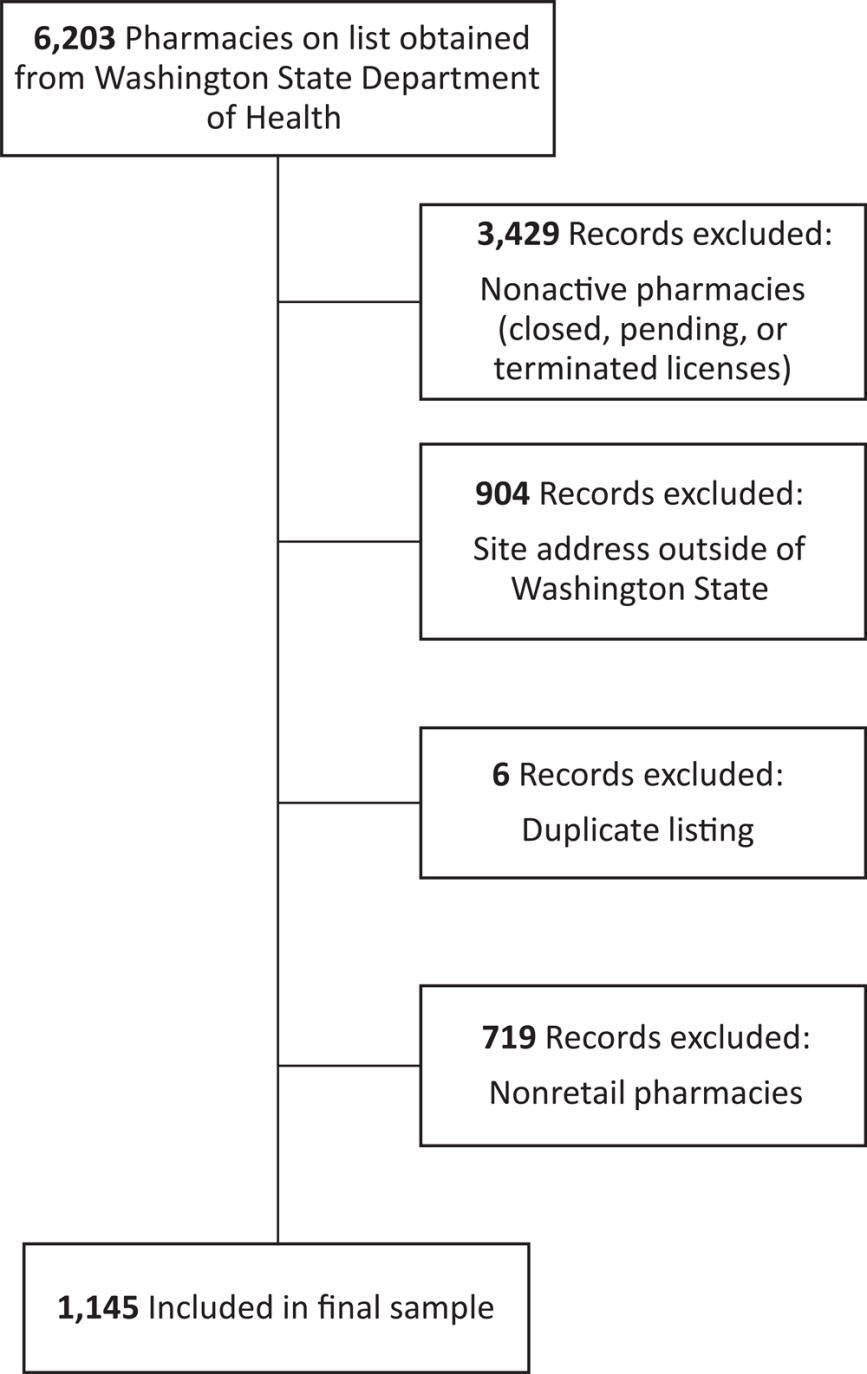
Flowchart of selection of pharmacies for study sample, Washington State, 2017.

**Pharmacy data.** We obtained business names, addresses, telephone numbers, and, if available, email addresses, for pharmacies from the Washington State Department of Health in August 2017 (N = 6,203) (Figure 1). We excluded from the sample pharmacies with pending, closed, or terminated licenses (55.3%, n = 3,429), pharmacies with a site address outside Washington State (14.6%, n = 904), and duplicate listings (0.1%, n = 6). To focus on the primary point of pharmacy contact for community residents, we excluded the following types of nonretail, noncommunity pharmacies (11.6%, n = 719): specialty clinics (n = 278), general clinics (n = 219), specialty pharmacies (n = 68), long-term care facilities (n = 33), compounding pharmacies (n = 26), jails (n = 21), hospitals (n = 20), urgent care clinics (n = 18), medical supply centers (n = 8), surgery centers (n = 8), imaging clinics (n = 7), pharmaceutical wholesalers (n = 4), dental clinics (n = 5), call centers (n = 2), a blood bank (n = 1), and a mail order pharmacy (n = 1). We did not exclude pharmacies in federally qualified health centers or community health centers. Two study team members reviewed exclusions independently; any discrepancies were individually reviewed and evaluated until consensus was achieved. The final sample consisted of 1,145 pharmacies.

In Washington State, 5 insurance carriers administer Medicaid insurance to enrollees. Each pharmacy in Washington State can hold a contract with none, some, or all 5 Medicaid insurance plans; Medicaid enrollees may choose any plan. We obtained the names, addresses, and contact information for pharmacies contracted with each Medicaid insurance plan from each health plan in September, October, and November 2017 through formal requests, direct download, or public record disclosure requests to the Washington Health Care Authority. Using business names, addresses, and telephone numbers, we manually matched data from Medicaid insurance plans to the list of actively licensed pharmacies from the Washington State Department of Health. Lists of insurance plans were sortable by county, city, or pharmacy name to facilitate matching. After matching, 2 study team members reviewed the entire list for accuracy. We then classified pharmacies by zip code. We created a dichotomous indicator for access to a Medicaid-contracted pharmacy (at least 1 pharmacy that was contracted with a Medicaid insurance plan in a zip code).

**Rural-Urban Commuting Area (RUCA).** We categorized zip codes into degrees of rurality by using the RUCA 3.10 framework classification Scheme 2 developed by a collaboration of the Health Resources and Service Administration’s Office of Rural Health Policy, the Department of Agriculture’s Economic Research Service, and the Washington, Wyoming, Alaska, Montana, Idaho (WWAMI) Rural Health Research Center (26). This scheme uses the geographic characteristics of population size, population density, and daily commuting patterns to establish 4 tiers of rurality: urban core (RUCA 1.0, 1.1), suburban (RUCA 2.0, 2.1, 3.0 and >100 residents/square mile), large rural (RUCA 4.0, 4.1, 4.2, 5.0, 5.1, 5.2, 6.0, 6.1 and >100 residents/square mile), and small town/rural (RUCA 7.0, 7.1, 7.2, 7.3, 7.4, 8.0, 8.1, 8.2, 8.3, 8.4, 9.0, 9.1, 9.2, 10.0, 10.1, 10.2, 10.3, 10.4, 10.5, 10.6 or not urban core with population density <100 residents/square mile) (26).

**Census data.** Characteristics of zip codes were derived from zip code–level data extracted from the 2017 American Community Survey 5-year estimates (27). We summarized data on the following community characteristics: total population (median and mean); mean percentage of population that was over age 65, nonwhite, Hispanic, and Medicaid-insured; and mean percentage with income below 200% of federal poverty level.

### Statistical analysis

We conducted all analyses in Stata/MP version 15.1 (StataCorp LLC). The primary outcome measure was access to a Medicaid-contracted pharmacy. We summarized zip code–level community characteristics across rurality by using descriptive statistics. We used a nonparametric test for trend (*nptrend*) across ordered groups to test for differences in access to a Medicaid-contracted pharmacy across levels of rurality. We also used χ^2^ tests to compare the proportion of zip codes in each level of rurality that had access to a Medicaid-contracted pharmacy.

We used a modified Poisson regression model (28) to examine the associations between rural–urban classification and access to a Medicaid-contracted pharmacy. Multivariable models included covariates relating to the following zip code–level characteristics: percentage of population over age 65, percentage of nonwhite population, percentage of Hispanic population, percentage of Medicaid-insured population, percentage of population with income below 200% of the federal poverty level, and total population size. We chose covariates a priori on the basis of differences in population characteristics between rural and urban areas that are likely to influence health services access and use (29). We assessed multicollinearity between covariates by using the variance inflation factor, with a factor above 10 indicating moderate to strong collinearity. We used ESRI ArcGIS version 10.5.1 to create a map that illustrates the geographic distribution of pharmacies and their contracting with a Medicaid insurance plan. For all analyses, we set significance at *P* < .05. 

## Results

Of the 1,145 pharmacies included in the final sample, most (78.3%, n = 896) were in urban areas; the remainder were located in suburban (7.8%, n = 89), large rural (8.1%, n = 93), and small town/rural (5.9%, n = 67) areas. Urban areas had a lower percentage of residents over age 65 and a higher percentage of nonwhite residents compared with other areas (Table 1).

**Table 1:** Zip Code Characteristics by Rurality Classification, Washington State, 2017^a^

Characteristic	Urban Core (n = 340)	Suburban (n = 120)	Large Rural (n = 69)	Small Rural (n = 177)	All Zip Codes (N = 706^b^)
Percentage aged >65	13.6	19.3	20.1	23.0	17.5
Percentage nonwhite	24.8	11.1	11.7	14.4	18.6
Percentage Hispanic	11.1	9.6	16.6	11.1	11.4
Percentage Medicaid-insured	16.0	16.4	16.8	21.3	17.4
Percentage with income below 200% of the federal poverty level	28.4	30.6	33.9	38.9	31.9
**Total population, no.**					
Median	24,972	3,950	6,472	1,340	9,785
Mean	25,530	7,510	11,238	2,749	15,359

a Data were obtained from the 2017 American Community Survey 5-year estimates and represent the means for zip codes in each rurality classification (27). All values are percentages, unless otherwise indicated.

b Census data did not link to zip code data for 8 zip codes (2 urban core, 2 suburban, and 4 rural).

Most pharmacies (91.6%, n = 1,049) were contracted with at least 1 Medicaid insurance plan. There were similar proportions of Medicaid-contracted pharmacies in urban core areas (92.3%, 827 of 896), suburban areas (91.0%, 81 of 89), and large rural areas (92.5%, 86 of 93); a significantly smaller percentage of Medicaid-contracted pharmacies were located in small rural areas (82.1%, 55 of 67) (χ^2^ = 8.6; *P* = .04).

**Figure 2:**
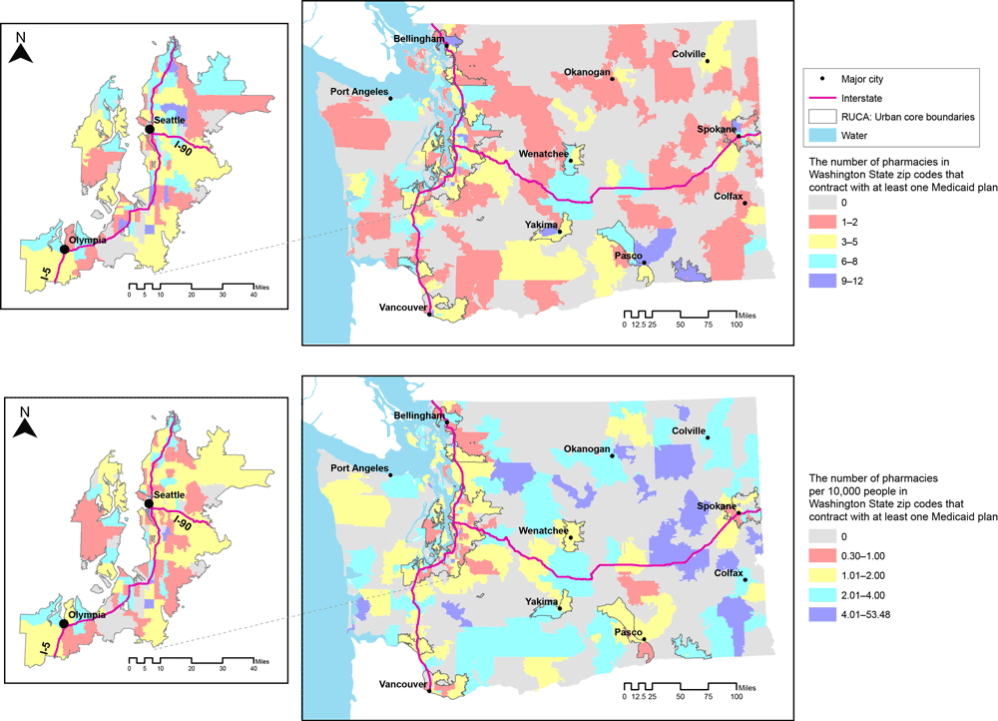
Geographic patterns of Medicaid-contracted pharmacies in Washington State, 2017, showing the number of pharmacies (top) and the number of pharmacies per 10,000 residents (bottom) in Washington State zip codes that contract with at least 1 Medicaid insurance plan. Inserts show the greater Seattle/Olympia area.

Overall, of 706 zip codes, 39.4% (n = 278) had access to at least 1 Medicaid-contracted pharmacy. Medicaid-contracted pharmacies were distributed throughout Washington State; we found larger proportions surrounding cities (Figure 2). The proportion of zip codes with a Medicaid-contracted pharmacy varied across urban (54.1%, 184 of 340), suburban (30.0%, 36 of 120), large rural (27.5%, 19 of 69), and small rural (22.0%, 39 of 177) classifications (χ^2^ = 61.7; *P* < .001). The nonparametric test for trend showed a significant decrease in the percentage of zip codes with a Medicaid-contracted pharmacy as rurality increased (Cuzick nonparametric trend test across ordered groups, *z* = −7.36; *P* < .001).

Unadjusted regression models showed a significant association between increasing rurality and limited access to a Medicaid-contracted pharmacy (Table 2). The multivariable (adjusted) regression model showed that the likelihood of access to a Medicaid-contracted pharmacy decreased significantly as rurality increased. Compared with urban core zip codes, small rural zip codes had a 36% lower likelihood of access to a Medicaid-contracted pharmacy, after adjusting for zip code characteristics (adjusted relative risk = 0.64; 95% CI, 0.46–0.91; *P* = .01). Large rural zip codes were also significantly less likely than urban core areas to have access to a Medicaid-contracted pharmacy, after adjusting for zip code characteristics (adjusted relative risk = 0.68; 95% CI, 0.49–0.95; *P* = .02). We found no evidence of multicollinearity; the variance inflation factor for all variables in the adjusted model was less than 10.

**Table 2:** Likelihood of Zip Code–Level Access to a Pharmacy Contracted With at Least 1 Medicaid Insurance Plan, Washington State, 2017^a^

Characteristic	Unadjusted Model		Adjusted Model	
	Risk Ratio (95% CI)	*P* Value	Risk Ratio (95% CI)	*P* Value
**Rurality classification**				
Urban core	1 [Reference]	—	1 [Reference]	—
Suburban	0.55 (0.41–0.74)	<.001	0.76 (0.58–1.01)	.06
Large rural	0.51 (0.34–0.76)	.001	0.68 (0.49–0.95)	.02
Small rural	0.41 (0.30–0.55)	<.001	0.64 (0.46–0.91)	.01
**Sociodemographic**				
Percentage aged >65	0.98 (0.97–0.98)	<.001	1.00 (0.99–1.01)	.55
Percentage nonwhite	1.01 (1.01–1.02)	<.001	1.00 (0.99–1.00)	.92
Percentage Hispanic	1.00 (0.99–1.00)	.92	0.99 (0.99–1.00)	.18
Percentage Medicaid-insured	0.99 (0.99–1.00)	.10	1.01 (1.00–1.02)	.13
Percentage with income below 200% of the federal poverty level	0.99 (0.99–1.00)	<.001	1.00 (0.99–1.00)	.33
Total population	1.00 (1.00–1.00)	<.001	1.00 (1.00–1.00)	<.001

a Adjusted model includes all listed covariates. Variance inflation factor for all variables <10.

## Discussion

Contemporary pharmacies have evolved today to expand beyond the traditional dispensing of medications and providing of over-the-counter products. Pharmacists continue to provide prescription monitoring and drug information and education; however, pharmacists now also offer a broad array of health care services. In rural areas, particularly in areas that no longer have a local primary care provider or clinic, pharmacies and pharmacists may be the only direct medical provider for rural residents. Pharmacies’ decision to contract with Medicaid can have a large effect on access to affordable medications, coordination of medications, and access to pharmacy services that are crucial for the prevention and management of chronic diseases.

Findings from our study illustrate disparities in access to pharmacy care for Medicaid recipients across the rural–urban continuum. After accounting for zip code–level characteristics, including measures of socioeconomic status and demographic characteristics, small and large rural areas of Washington State were significantly less likely than urban core areas to have access to a pharmacy that was contracted with at least 1 Medicaid insurance plan. This disparity in access could negatively affect prescription adherence and access to critical public health services now offered at pharmacies.

Policy efforts aimed at promoting and reducing barriers to telepharmacy may help to improve access to pharmacy services in medically underserved rural areas (30). Researchers who have developed and tested telepharmacy interventions have reported promising findings on patient self-management of chronic conditions and that rural patients are largely satisfied with interacting with pharmacists remotely (31). Telepharmacy has also been shown to decrease health care use among rural veterans at high risk for adverse drug events and medication reconciliation discrepancies (32). Despite the potential for improving access to care in medically underserved areas, only about half of the states in the United States have passed legislation allowing telepharmacy, and regulations vary by state (33). Addressing issues such as reimbursement, licensing, and data security (33) will be important for expanding pharmacy services in the United States.

In addition to pharmacy closures, that more than 1 in 6 pharmacies in small rural areas are not contracted with a Medicaid insurance plan and that nearly 80% of rural zip codes lack access to a Medicaid-contracted pharmacy, as shown in our study, generates increased concern about limited access to pharmacy services in many rural communities. The implementation of Medicare Part D and associated challenges with reimbursement, payment, and claims management have been implicated in reduced revenues for and closures of independently owned pharmacies, especially pharmacies in rural areas (7,34–36). As a by-product of the challenges created by Medicare Part D expansion, Medicaid patients may face a lack of access to critical services. Policy efforts targeting improved access to pharmacy services may require incentive allocations for Medicaid-contracted pharmacies based on geographic location. Pharmacists in both rural and urban areas have reported that lack of reimbursement is one of the top barriers to expanding the delivery of public health services (37).

Our study has several limitations. Many health plans encourage enrollees to use mail order pharmacies. Medicaid enrollees can receive recurring prescriptions and refills by mail rather than in local pharmacies. Therefore, although the results of our study may be less applicable to prescription services for long-standing chronic conditions, they are relevant for dose and formulary changes, as well as for acute conditions that require timely access to prescriptions. Community retail pharmacies serve an important role beyond ensuring timely access to prescriptions.

We did not determine the kinds of services offered at each pharmacy in our study. Some community retail pharmacies in rural parts of Washington State may provide only traditional services and not ancillary public health services. Additionally, we included pharmacies associated with community health centers in our study; however, we excluded pharmacies in general and specialty clinics because we could not determine whether they limited their services to clinic patients, which such clinics often do. We also could not determine why each pharmacy chose not to contract with a Medicaid insurance plan. Pharmacies are not mandated to contract with Medicaid insurance, and the low reimbursement rates and administrative burden may lead pharmacies to forgo contracting with this insurer. Future research examining these pharmacy-level factors may provide additional insight into the trends observed in our study.

Community retail pharmacies provide vital prescription services to people needing acute and chronic treatment as well as disease management and health promotion services. Our study showed that although 91.6% of pharmacies in our study were contracted with Medicaid, geographic areas located outside urban core centers were significantly less likely to have access to a Medicaid-contracted pharmacy. In small rural areas, nearly 80% of zip codes did not have access to a Medicaid-contracted pharmacy, compared with nearly half of urban core zip codes. This disparity in access to health care at pharmacies can place an undue burden on residents in rural areas. Barriers in accessing traditional and ancillary pharmacy services should be minimized for publicly insured individuals who live in rural and medically underserved areas of Washington State.
